# Noise-Robust Multimodal Audio-Visual Speech Recognition System for Speech-Based Interaction Applications

**DOI:** 10.3390/s22207738

**Published:** 2022-10-12

**Authors:** Sanghun Jeon, Mun Sang Kim

**Affiliations:** Center for Healthcare Robotics, Gwangju Institute of Science and Technology (GIST), School of Integrated Technology, Gwangju 61005, Korea

**Keywords:** deep learning, audiovisual speech recognition, lipreading, multimodal interaction, edutainment, virtual aquarium

## Abstract

Speech is a commonly used interaction-recognition technique in edutainment-based systems and is a key technology for smooth educational learning and user–system interaction. However, its application to real environments is limited owing to the various noise disruptions in real environments. In this study, an audio and visual information-based multimode interaction system is proposed that enables virtual aquarium systems that use speech to interact to be robust to ambient noise. For audio-based speech recognition, a list of words recognized by a speech API is expressed as word vectors using a pretrained model. Meanwhile, vision-based speech recognition uses a composite end-to-end deep neural network. Subsequently, the vectors derived from the API and vision are classified after concatenation. The signal-to-noise ratio of the proposed system was determined based on data from four types of noise environments. Furthermore, it was tested for accuracy and efficiency against existing single-mode strategies for extracting visual features and audio speech recognition. Its average recognition rate was 91.42% when only speech was used, and improved by 6.7% to 98.12% when audio and visual information were combined. This method can be helpful in various real-world settings where speech recognition is regularly utilized, such as cafés, museums, music halls, and kiosks.

## 1. Introduction

Recently, with the rapid development of deep learning, the educational, experiential, and auxiliary situations in which various deep learning technologies are applied have increased [1,2,3,4]. In particular, in terms of edutainment, various recognition technologies based on deep learning are being developed to recognize interactions such as speech, gestures, eye and head tracking, and even physical objects. The term edutainment, a portmanteau of education and entertainment, is an approach designed to be simultaneously educational and fun, and is widely used in robot platforms, museums, science centers, and aquariums [5,6,7]. Edutainment techniques that simultaneously provide both education and fun reportedly have an effect on learning outcomes, and numerous systems have consequently been developed [3,8,9,10]. They are also useful for interacting with the elderly [1].

**Figure 1:**
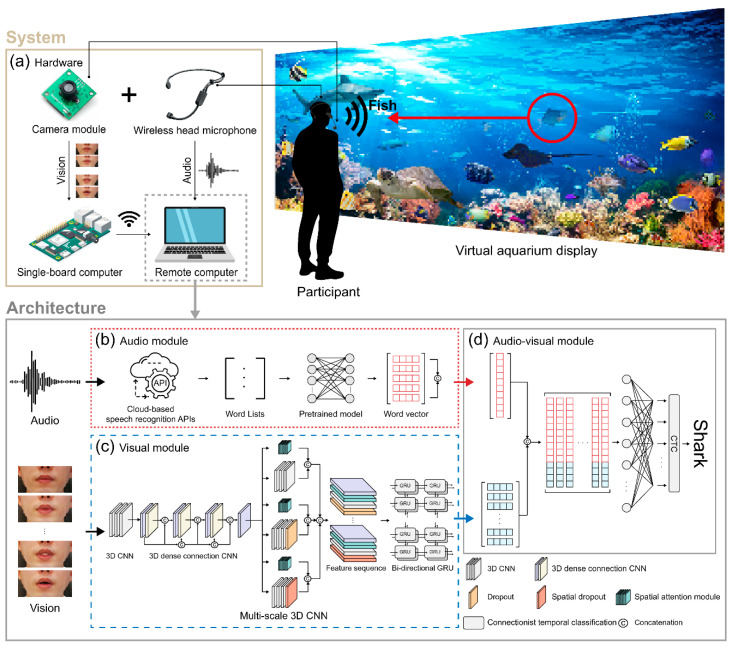
Composition of the proposed system and an example usage scenario. (**a**) Hardware components of the system and a virtual aquarium interaction usage scenario; (**b**) audio module for generating word vectors; (**c**) visual extraction module for extracting feature vectors; (**d**) audiovisual module for classification.

Speech is one of the most commonly used interaction-recognition techniques in edutainment-based systems, and is a key technology for smooth educational learning and interaction between the system and the user [10,11,12,13,14]. Speech involves the perception of both auditory and visual information and is the most commonly used human engagement and communication mode. Janowski et al. [15] compared gestures and speech experimentally for four separate interaction tasks (navigation, selection, dialogue, and manipulation) in a virtual environment. They reported that in object manipulation, both speech and gestures were preferred. However, despite the increasing preference for interaction using speech, the application of interaction using speech to real environments is quite limited owing to the various noise disruptions that can occur in real environments. Therefore, core technology that is robust to difficult acoustic scenarios and various noises in real environments is essential. Consequently, in this study, an audio and visual information-based multimode interaction system is proposed that enables virtual aquarium systems that use speech to interact to be robust to ambient noise. Figure 1 gives a conceptual overview of the proposed system being utilized for interaction via speech in a virtual aquarium.

Multisensory integration has an important effect on human communication, and each sensory organ transmits specific sensory information [16]. The human nervous system is organized into multiple nonoverlapping sensory organs and uniquely processes the received input, which enables it to sense very reliably. Depending on the received input information, information from different senses can be connected to each other, thereby synergistically enhancing the ability to recognize and evaluate [17,18].

People can improve their ability to understand speech in noisy environments or where it is otherwise difficult to understand speech by detecting the movements of the tongue and teeth and the area around the speaker’s lips. In other words, when the speech signal is not clear, visual speech information has a significant positive effect on speech comprehension [18,19,20,21,22,23]. As such, humans tend to process visual information first when visual and auditory information are received simultaneously. This phenomenon is called “synesthesia” or the “McGurk effect” in cognitive psychology [24]. Therefore, we judge many things visually to the extent that the sound we hear depends on what we see. For example, if people see a person’s face expressing “ba” and hear the sound “ga,” many of them will infer the third sound “da” that combines the two. The fusion approach can contribute to robust recognition of speech interactions in real life and overcome the problem of auditory and visual ambiguity of words with similar pronunciation in noisy environments.

This work offers a noise-robust system for use in virtual aquariums by integrating a deep neural network-based end-to-end visual speech recognition architecture with an open cloud speech recognition (OCSR) API system (Figure 1). The performance of this system is superior to that of single-mode systems that use either audio or visual speech recognition technologies. For audio-based speech recognition, a pretrained model is used to express word vectors for a list of words that have been identified by the speech API. Meanwhile, vision-based speech recognition uses a new deep neural network-based lipreading architecture consisting of end-to-end neural subnetworks. We consecutively combined three 3D convolutional neural networks (CNNs) for feature sequence extraction, shortening the training time by reducing the number of parameters, and referring to the existing 2D DenseNet [25] to suppress overfitting. The 3D densely connected CNN is composed of the components of a multichannel 3D CNN to extract the multichannel features of different levels. A bidirectional gated recurrent unit (GRU) followed by a linear layer is used to overcome the scarce visual information caused by the time-series input data and to obtain specific image features. A vector matrix is formed by connecting the word and feature vector output to the audio- and visual-based model. By inserting a SoftMax layer at each time step and then applying the connectionist temporal classification (CTC) loss function [26] to all time steps, the concatenated vector matrix is trained to acquire predictive words.

We also compared the accuracy and efficiency of the proposed system with existing single-mode techniques for extracting visual features and multiple OCSR APIs from the collected dataset. The results of extensive evaluations verified that the proposed system achieved faster convergence speed, higher computational efficiency, and superior performance. Thus, our system, which integrates the existing OCSR API system with an end-to-end lipreading architecture using visual information, is widely utilizable for diverse speech-based interaction settings such as cafés, music halls, and virtual aquariums.

The main contributions of this paper are as follows:

- We propose a novel audiovisual speech recognition system using multimode interaction for virtual aquarium applications that is robust to ambient noise;
- We compare the accuracy and efficiency of the proposed system with those of existing single-mode techniques for extracting visual features and multiple OCSR APIs from a collected dataset;
- We show that the proposed system provides faster convergence speed, higher computational efficiency, and superior performance compared with the existing OCSR API system.

The remainder of this paper is organized as follows. Section 2 of this paper gives an overview of research related to this study. Section 3 provides the details of each element of the proposed system. Section 4 provides information on the datasets collected, data augmentation techniques, and experimental setup. Section 5 presents the training procedure, convergence rate, optimization, and performance evaluation results of the proposed system. Section 6 and Section 7 discuss the experimental results according to the research objectives, suggest directions for future research, and provide our conclusions.

## 2. Related Work

Google has released an open cloud-based speech API [27] along with various functions and application scenarios for finance, automobiles, and hospitals. It has advanced capabilities such as distinct speaker identification, automated speech language detection, data logging, and speech-to-text conversion with multiple channels, as well as support for over 125 languages. IBM’s Watson AI service [28] supports 11 languages and provides services such as dialogue dialing of the speaker and word extraction and filtering, along with the ability to customize specific volume conditions by applying the system according to the usage environment. However, to use the pretrained language model, there is also a difficulty, in that it needs to be tuned to the customer management domain, and this requires adding a new corpus. In addition, Microsoft offers the speech-to-text capabilities of the Speech Service, part of Azure Cognitive Services [29], as a cloud service package. According to the official website, the Speech Service includes real-time speech synthesis, asynchronous synthesis of long audio, prebuilt neural network speech, and viseme technology. Xiong et al. [30] reported that it achieved human-level accuracy for the first time in a switchboard test in 2017. Amazon Alexa [31] is an artificial intelligence (AI) smart personal assistant with a speech-activated platform that enables voice interaction and Q&A. Alexa has various other capabilities, including the ability to play music, set alarms, and obtain weather information. It can also be used to operate smart home technology devices.

Owing to rapid improvements in the field, deep learning has lately delivered good performance in various applications in diverse research domains, including VSR systems. Deep learning-based algorithms outperform traditional prediction methods. For example, the systems proposed by Ji et al. [32] and Petridis and Pantic [33] distinguish different visemes by integrating the traditional approach with a CNN, and add time information after obtaining the CNN output using the hidden Markov model framework. Wand et al. [34] and Cooke et al. [35] integrated histograms of oriented gradients (HoG) with long short-term memory (LSTM) to evaluate a GRID benchmark dataset consisting of short phrases. In addition, the LSTM classifier was trained on the OuluVS and AVLetters datasets [35] using the discrete cosine transform, and word prediction evaluation was performed. Noda et al. [36] applied the sequence-to-sequence (seq2seq) model to lipreading. The model has a deep speech recognition architecture capable of recognizing and predicting the output of full input sequences. In addition, performance evaluation was performed on a benchmark dataset composed of real words by integrating all the audiovisual information.

Assael et al. [37] introduced LipNet, an end-to-end deep learning model, and it was trained and its performance evaluated on the GRID corpus, a sentence-level dataset. The GRID corpus was divided into overlapped and unseen-speaker database structures, and it achieved word error rates of 4.8% and 11.4%, respectively. However, the evaluation was performed with the same database, and the experienced human lip reader had a low success rate of 47.7%. Fenghour et al. [38] presented a deep learning network model for viseme-to-word translation that used an attention-based GRU and enhanced the performance of predicting spoken sentences by reaching a word accuracy of 79.6%. Li et al. [39] proposed an efficient two-stream model for learning dynamic information. The model extracts static characteristics from a single frame and dynamic information between multiple frame sequences using two distinct channel capacity CNN streams. Utilizing a more effective convolutional structure for each component in the front-end model yielded an 8% improvement. Xu et al. [40] implemented and evaluated a digit sequence prediction and an architecture similar to the CTC cascaded model on audiovisual datasets. Deep learning-based approaches are more resilient to huge data and visual ambiguity than conventional information-extraction techniques, and they can extract more precise, detailed, and accurate information from audiovisual data.

The evaluation and comparison of the recognition performance of previously available OCSR APIs have been the focus of several studies [41,42,43]. Contrastingly, the goal of this study is to enhance the interactive performance in a virtual aquarium by incorporating visual information into the already-existing OCSR API system. Compared with the traditional OCSR API system, the method performs better recognition and reduces the error rates in noisy environments. Consequently, in this work, we present an interaction model that, in contrast to the current interaction approach that only utilizes auditory information, employs audiovisual information based on deep learning and uses a system with a low error rate even in noisy environments.

## 3. Architecture of the Proposed System

Our proposed deep learning audiovisual speech recognition interaction system, which is integrated with the open cloud-based speech API, is shown in Figure 1. As shown in Figure 1b, human speech is input and the recognized words are converted into word vectors using a pretrained word embedding model. Simultaneously, as shown in Figure 1c, a face video matching the human speech is received and extracted as a sequence feature vector. Subsequently, the audiovisual module (Figure 1d) predicts a word or sentence by integrating the word and sequence feature vectors output from the audio and visual modules (Figure 1b,c).

### 3.1. Audio Module

In the audio speech recognition module, the speech API receives the user’s speech via a microphone and sends it to an open cloud-based recognition system. The open cloud-based speech recognition API is a publicly available API for developing applied speech recognition systems. It has a speech recognition engine created by collecting large amounts of speech data via a cloud computing service and learning on large amounts of data with high-performance computing. Cloud firms provide these speech recognition engines so that anyone can use them without difficulty via the voice recognition Open API, saving significant amounts of development time, effort, and expense.

**Figure 2:**
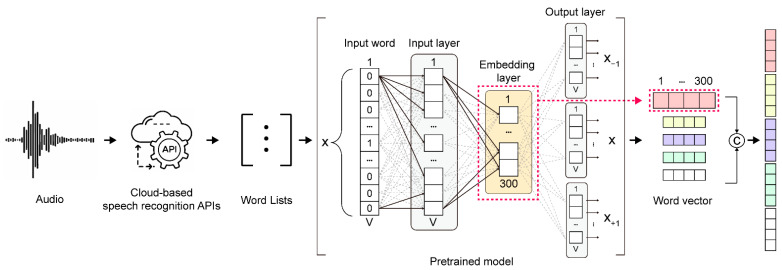
Block diagram of the proposed audio speech recognition module.

Figure 2 is a block diagram of the proposed audio speech recognition module; it utilizes two general algorithms. The human speech is input via a local device such as a microphone, and the recorded speech is delivered to an open cloud server provided by a commercial engine, Microsoft Azure, for further processing [44]. Through speech recognition by the OCSR API, which is closed-source, it is possible to quickly implement a user-optimized speech recognition system and has the usability advantage of being immediately applicable to various fields. Therefore, developers using application speech recognition systems should choose the appropriate OCSR API based on the capabilities of the system. In addition, the performance of the OCSR API is constantly updated by many companies that provide API services, and it depends on the study date and the type of training data. Therefore, we used the Microsoft Azure API, which has been proven to be superior in the results of previous studies [45]. In addition, the existing speech API can be replaced if another speech API with better performance is released and is not affected by performance changes over time. The word lists output through the OSCR API are output as individual word vectors using Google’s pretrained Word2Vec embedding model. These word vector representations may have hundreds of dimensions in the Word2Vec model, also known as word embeddings [46,47]. For academic use, Google offers a Word2Vec model that has been pretrained on 100 billion words from the Google News corpus, producing 3 million 300-dimensional word embeddings. Therefore, a list of words is output, transformed into a 300-dimensional vector, and concatenated into a single vector.

### 3.2. Visual Module

**Figure 3:**
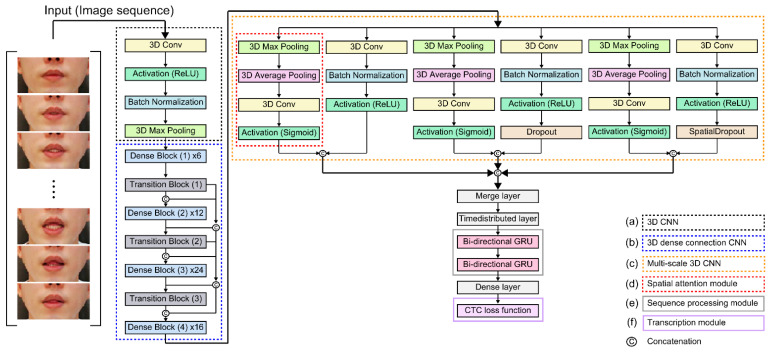
Detailed schematic of the proposed visual speech recognition module.

Figure 3 is a detailed schematic of the proposed visual speech recognition module. It consists of three modules: feature extraction, sequence processing, and transcription. The feature extraction module consists of three CNNs: 3D CNN to extract fine motion around the lip, 3D dense connection CNN to reduce model parameters and prevent overfitting, and multiscale 3D CNN to extract rich features with different level features. The sequence-processing module uses Bi-GRU to comprehend a wide sequential feature context, and the transcription module combines the local self-attention mechanism in a cascaded approach to compensate for the shortcomings of the CTC loss function, focusing only on local information in the nearby frame.

#### 3.2.1. Feature Extraction Module

**Figure 4:**
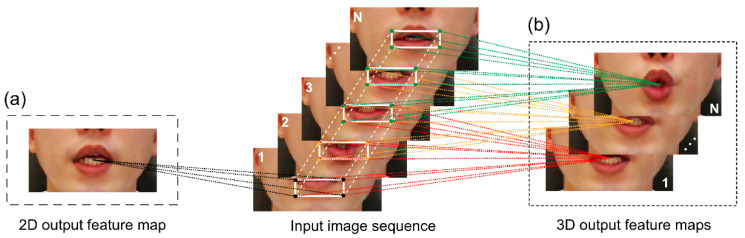
(**a**) Two-dimensional convolution operation. (**b**) Three-dimensional convolution operation.

Figure 4a shows that the 2D CNN collects encoded information about single-image data and transforms the information into a 2D feature map to calculate spatial-dimensional features. However, a 2D CNN cannot extract motion information in consecutive frames extracted from video.

As shown in Figure 4b, we need a 3D CNN that can simultaneously calculate spatial and temporal dimensional features to detect various peripheral information such as tongue and tooth movement information around the lips. The 3D CNN is a widely used technique to detect spatial and temporal information in time-series sequence data and has been proven to be effective in extracting spatial and temporal information in numerous studies [32,37]. In this study, CNN layers comprising 64 3D kernels of size 3 × 7 × 7 were constructed to extract and encode visual feature information into input sequential lip data, and combined with the batch normalization layer and ReLU. Subsequently, the spatial scale of the 3D feature map was reduced by connecting the max-pooling 3D layer (Figure 3a). The details of the proposed model hyperparameters are presented in Table A1.

**Figure 5:**
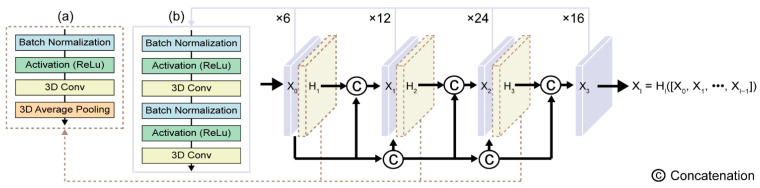
Detailed 3D dense-connection CNN architecture. (**a**) Three-dimensional transition layer; (**b**) three-dimensional dense block.

Following the 3D CNN, a 3D dense-connection CNN is used to reduce the parameters of the model to save processing resources and effectively prevent overfitting. With this method, relationships between several linked layers are generated, facilitating network depth, vanishing gradients, and full functional utilization (Figure 5). We extend the 3D volumetric feature extraction task by referring to the existing 2D densely connected CNN structure composed of the *l*-th layer of the nonlinear transformation $H_{l}$. The output of the *l*-th layer of the existing 2D structure can be represented by $x_{l}$ (Equation (1)), where $x_{0},\;x_{1},\;\ldots ,\;x_{1-1}$ are generated in the previous layer and […] denotes the concatenation operation [24].

(1) $$x_{l}=H_{l}\left(\left[x_{0},\;x_{1},\;\ldots ,\;x_{1-1}\right]\right)$$

This approach utilizes two modules: a 3D transition layer module and a 3D dense block module. The feature maps processed in the 3D CNN are reduced by the bottleneck layers (Figure 5b) and then multichannel feature volumes are integrated. As the previous feature information still exists, subsequent layers are applied only to a few feature volumes, and the hyperparameter ta controlling the degree of compression is also included in the transition layer (Figure 5a) to increase compressibility. Thus, reduced growth rates can be achieved by using bottlenecks and transition layer tiers in succession. The dense block structure is doubly connected in the following order: batch normalization (BN) layer, activation function (ReLU), and 3D convolutional layer (Figure 5b). The transition layer connected after the dense block structure has the same structure as the dense block structure, and an average-pooling 3D layer of 2 × 2 × 2 is additionally connected (Figure 5a). The 3D convolutional layer used for the dense block structure is 3 × 1 × 1, and is composed of 3 × 3 × 3 3D convolutional layers of the transition layer.

**Figure 6:**
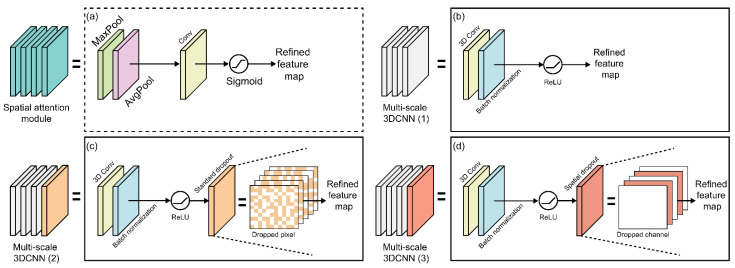
Multilayer 3D CNN architectures: (**a**) spatial attention module; (**b**) first architecture; (**c**) second architecture with standard dropout approach; (**d**) third architecture with spatial dropout approach.

By implementing them in various sizes and depths, we coupled multiscale 3D CNNs to extract various layers of spatial and visual information. In the multiscale 3D CNN, multiple convolutional layers of different level sizes can generate different level features based on different depths and filters, and this strategy can be used to extract richer feature information with a layered approach (Figure 3c). The proposed multiscale 3D CNN architecture is shown in Figure 6; it is divided into four structures. The three modules are composed of different kernel sizes based on the structure of Figure 6b. The first module, Figure 6b, connects to a 3D convolution layer with a 3D kernel size of 32 in order of batch normalization and activation functions (ReLU). The second module (Figure 6c) and third module (Figure 6d) add standard and spatial dropouts with 3D convolution layers of different 3D kernel sizes of 64 and 96, and then add activation functionality (ReLU).

The function of the standard dropout is to prevent strongly correlated activations in the image feature map by randomly dropping pixels, thereby preventing overfitting and overtraining, which affect the CNN performance improvement. Therefore, it plays an important role in small benchmark datasets compared to large datasets such as image classification datasets [48]. The spatial dropout of the third module outperforms and performs in strongly spatially correlated image classification by dropping the corresponding channel rather than pixels [49,50]. It is particularly effective in extracting the fine motion features of the lips, teeth, and tongue with strong spatial correlation. Additionally, to focus on the location of the information section and complement the attention channel, all three modules are combined with spatial attention modules of the same structure (Figure 6a). The spatial attention module focuses on utilizing interspace interactions to better select the most identifiable and useful portions of the input image [51]. It initially runs max-pooling and average-pooling operations along the channel axis and then connects them to create an efficient feature descriptor that calculates spatial attention. Therefore, the output of each multiscale 3D CNN and spatial attention module is merged and concatenated.

#### 3.2.2. Sequence Processing Module

Because the feature extraction module extracts only fixed short viseme-level features, it is difficult to distinguish the longer context information of random-length time-series input data. We use a GRU that learns to propagate and control the flow of time-series data information using update and reset gates [52]. The GRU is derived from the LSTM unit that determines which information should be conveyed and which should be ignored, allowing for the use of update and reset gates to solve the gradient loss problem. A bidirectional GRU is configured with the feature sequence of the feature extraction module as input to provide forward and backward information so that both networks can obtain rich information.

#### 3.2.3. Transcription Module

We use the CTC method, which does not require end-to-end alignment of deep neural networks and parameterizes the distribution of a label token sequence using a loss function. The marginal distributions created at each time step of the temporal module are conditionally independent of CTC. This is because it restricts the use of autoregressive connections to handle the inter-time-step dependencies of the label sequence. When the probabilities of the language model are ambiguous, the CTC models are decoded using a beam search approach to restore label temporal dependency.

## 4. Experimental Evaluation

### 4.1. Dataset, Data Preprocessing, and Augmentation

To evaluate the proposed model, we constructed a new dataset for the interaction of the virtual aquarium by referring to the Word Choice part of the most used speech recognition commands in IoT or real life in Google Speech Command Dataset V2 [53]. Currently, because most benchmark datasets are audio-based or consist of datasets used in real-world applications, datasets for the interaction of virtual aquariums are insufficient. Therefore, we constructed the dataset ourselves to evaluate the proposed model (Table 1).

To perform aquarium interaction in a virtual environment, data were collected by dividing them into two categories—an operation command to manipulate the system and a command to control objects in the virtual environment. As the unit for operating the system is not a complete sentence but a word or a short phrase, the collected words are generally useful as commands for automobile and robot applications [53].

**Figure 7:**
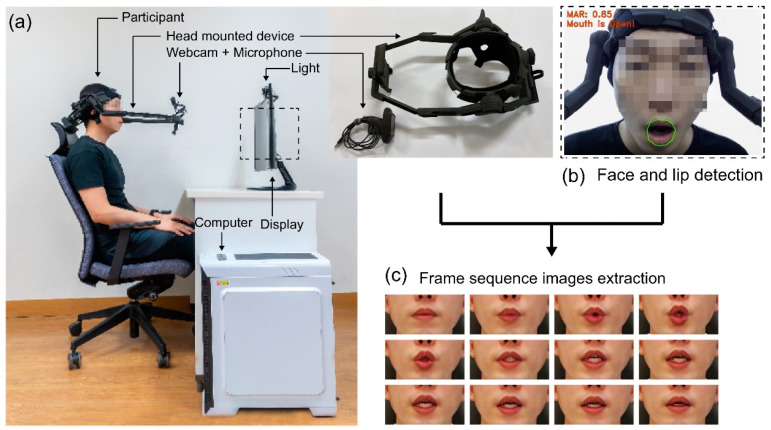
Data-recording environment and image extraction procedure: (**a**) Data-recording environment; (**b**) during data collection, the participant’s face information is visible to the camera, and the green line demarcates the lip detection area; (**c**) frame sequence images extracted from the acquired video.

**Figure 8:**
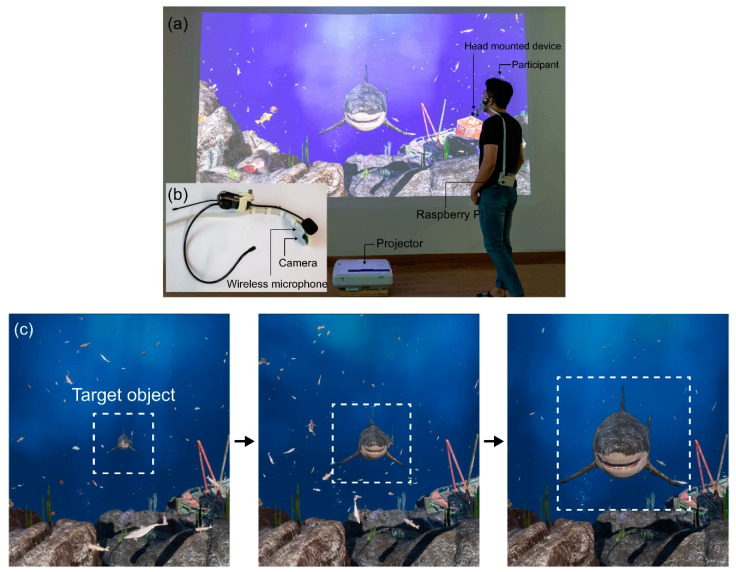
(**a**) Participant in the real-world interaction environment; (**b**) head-mounted device design; (**c**) target object approaching as a result of speech interaction.

For data collection, 40 participants (20 males and 20 females; average age: 29.14 years) who were familiar with speech recognition equipment were recruited and ultimately compensated with gift certificates worth USD 20. To ensure balance in the data, 10 males and 10 females were native English speakers, and the other 10 males and 10 females were bilingual advanced English-speaking participants. The participants wore a head-mounted device that combined a webcam with a camera and microphone (Figure 7). Each participant stared at the display (Figure 8b) located in front and repeated the 54 keyword lists 100 times at 2 s intervals in sequence.

The collected video underwent frame extraction and was output as shown in Figure 8c. We collected a total of 216,000 video clips for audio and video information. We used a Logitech webcam (C920 HD PRO WEBCAM) with the following specifications for data collection: video resolution of 1920 × 1080 (FHD), frame rate of 30 fps, and a stereo microphone. The face-facing webcam fixture was made with a 3D printer. The video data were recorded for 2 s with a resolution of 640 × 480 pixels at 30 fps, and the audio was stereo with a sample rate of 44,100 Hz.

We trained the proposed model on data fired 100 times per class and divided the data into training and validation sets in a 7:3 ratio. During the data collection process, there were participants whose pronunciation became weaker as the number of experiments increased because of the repetitive speech. Therefore, to prevent training overfitting from the problem of poor pronunciation, we used the most focused early (20–30) utterances, middle (50–60) utterances, and finally some of the utterances when concentration was lowest among the 100 utterances for validation. The utterances were selected and divided into training and validation sets in a 7:3 ratio. Specifically, the training dataset comprised utterances 1–9, 10–20, 31–50, 61–80, and 91–100, and the validation dataset comprised utterances 21–30, 51–60, and 81–90.

We used the Dlib [54] face detector with a HoG feature-based linear classifier to search the target region as a preliminary step for extracting human lip information in the data preprocessing step. The Dlib library, which can utilize image processing and different machine learning methods, is a general-purpose cross-platform software library developed in C++ that can use HoG features or a trained CNN model for face recognition. To create a bounding box around the mouth, the detected output was presented as (*x*, *y*) diagonal edge coordinates. Then, 68 landmarks and the same lip point as the data obtained from the training dataset with the online Kalman filter iBug [55] program were extracted. Using affine transformation, we extracted pictures with a size of 100 × 50 pixels from the target face region that was extracted in relation to the mouth’s center region. We then normalized the RGB channels of the entire training set so that the mean and unit variance were both zero. Furthermore, because—unlike other image classification data—overfitting may occur owing to the small amount of data, data augmentation was performed to prevent overfitting [41]. All models were trained and assessed using the same dataset pretreatment and augmentation approaches, and normal and horizontally mirrored image sequences were used for data augmentation throughout the training phase.

### 4.2. Implementation

The diagram on the left side of Figure 8 is a schematic of the overall system for processing the respective audio and visual information. Figure 8a shows a participant interacting with a virtual aquarium through speech commands. The participant wears a device that combines a wireless microphone and a small camera, as shown in Figure 8b, and controls the target object (e.g., a shark) via speech commands. A single-channel wireless head microphone is used to acquire the experimenter’s voice information, which is wirelessly transmitted to a remote computer. Simultaneously, visual information is acquired using a camera module attached to the wireless head microphone, and the connected single-board computer, Raspberry Pi, uses the robot operating system (ROS) to wirelessly transmit the visual information to the remote computer.

We used the CTC decoder with the beam search method to evaluate the character accuracy rate (CAR) of the proposed model. The evaluation environment consisted of an Intel^®^ CoreTM i7-7700K CPU running the Linux Ubuntu 18.04 LTS operating system, with 32 GB RAM, and an NVIDIA GeForce RTX 2080-Ti GPU. In addition, Keras based on the TensorFlow backend was used to evaluate the performance of the CTC decoder. Table A1 provides information on the architecture utilized for the evaluation. The specific requirements for each layer of the proposed model are listed as hyperparameters.

For model optimization during training, three evaluations were performed: optimization, batch size, and learning rate. To perform model optimization, AdaDelta [56], AdaGrad [57], adaptive moment estimation (Adam) [58], stochastic gradient descent (SGD) [59], RMSprop [55], AdaMax, and Nadam [59] were evaluated with a mini-batch of four and a learning rate of 0.0001. After performing model optimization, the model was executed to determine the optimal mini-batch size (0.1, 0.001, 0.000.1, 0.00001, or 0.000001) and learning rate (4, 8, 16, 32, or 64). A maximum batch size of 64 was used because of the limitations of our GPU memory.

We performed data collection and performance evaluation while considering factors such as lighting that could have a detrimental effect on the proposed system. The performance evaluation was conducted in an environment different from the conventional data collection environment, and for the evaluation, participants used a head-mounted webcam device (Figure 8). The performance evaluation environment was a natural environment without any controls, i.e., an environment with natural light and noise. The examination was separated into three sections: audio only, visual only, and combined auditory and visual information. Furthermore, none of the individuals that engaged in data collection during the performance evaluation stage participated in the generalized performance evaluation.

While taking into account external elements impacting the accuracy, such as illumination, the proposed system was assessed in an environment that differed from the data gathering setting. The participants used a head-mounted device with a webcam and did the evaluation while standing 1.5 m away from the virtual aquarium screen during the performance evaluation stage (Figure 8). In the experiment room, it was performed with normal lighting and noise, and only participants who had not taken part in the data collection were selected. The multimodal technique used in this investigation was divided into three categories: audio-only, visual-only, and audiovisual speech.

### 4.3. Performance Evaluation Metrics

We examined the learning loss, batch size, and optimization to assess the learning state throughout the proposed model’s training process, and we utilized the character error rate to assess the accuracy. Specifically, we converted the error rate measure to a percentage by calculating the total edit distance and compared the predicted text with the original text. In addition, we used a confusion matrix approach for visualization of the predicted data. The CAR for accuracy evaluation consists of five variables (C, S, D, I, N). They signify the characters (C), false prediction characters (S), number of deleted characters (D), unselected characters (I), and total number of correct characters (N). The proposed model is a maximum-probability prediction method that performs the CTC beam search technique. The CAR equation is expressed as follows:

(2) $$\mathrm{CAR}\;\left(\%\right)=100-\left(\frac{C_{S}+C_{D}+C_{I}}{C_{N}}\right)\times 100$$

To visualize and analyze the predicted data after learning, we used a confusion matrix, which is commonly used to summarize the performance of a classification model. The matrix compares the real findings with the test data to the number of properly and incorrectly categorized samples. Using the confusion matrix as a tool for evaluation provides the benefit of allowing a more thorough analysis in the event that the dataset is imbalanced, as opposed to depending solely on the fraction of correctly recognized samples (which could cause misleading conclusions). In Table 2, the confusion matrix for two classes is presented.

The true-positive, false-positive, true-negative, and false-negative values in the confusion matrix served as the basis for the algorithms’ performance parameters. Precision, recall, and F1-score were among the metrics computed. Classwise accuracy, recall, and F1-score were used to assess the classification models. These performance metrics were calculated using the following formulas.

Precision denotes the outcome of a scenario that is thought to be positive:

(3) $$\mathrm{Precision}=\frac{\mathrm{TP}}{\left(\mathrm{TP}\;+\;\mathrm{FP}\right)}$$

Recall reveals the estimation of the success of positive situations:

(4) $$\mathrm{Recall}=\frac{\mathrm{TP}}{\left(\mathrm{TP}\;+\;\mathrm{FN}\right)}$$

The F1-score, which represents the overall classification accuracy, is calculated as the harmonic average of recall and precision:

(5) $$F1-\mathrm{score}=\frac{2\times \mathrm{Precision}\;\times \mathrm{Recall}}{\left(\mathrm{Precision}\;+\;\mathrm{Recall}\right)}$$

Additionally, noise was generated with a signal-to-noise ratio (SNR) approach using the gathered source data to assess the trained model’s quantitative performance. Both the commonplace noise found in daily life and the multichannel acoustic noise database (DEMAND) served as the source of noise data for the noise-generation process. DEMAND comprises different types of noise from eight environments (parks, corridors, restaurants, stations, cafés, plazas, cars, living) and ambient noise (Table 3).

## 5. Results

### 5.1. Training Procedure and Convergence Rate

**Figure 9:**
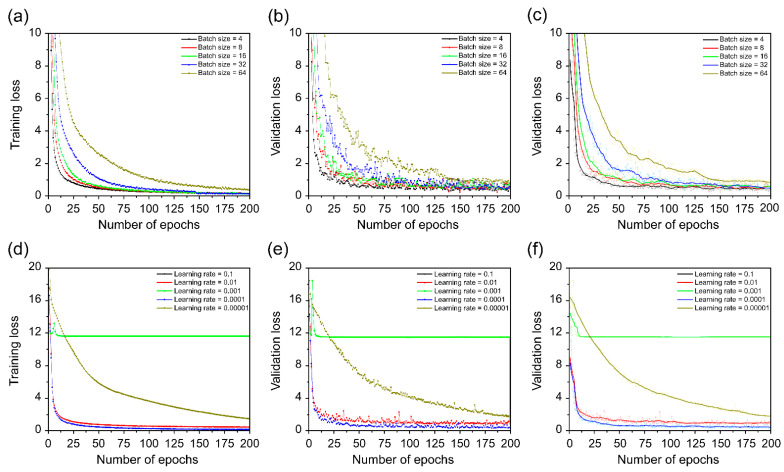
Training and validation loss of the collected dataset. (**a**) Different training batch sizes; (**b**) different validation batch sizes; (**c**) different validation batch sizes using moving average; (**d**) different training learning rates; (**e**) different validation learning rates; and (**f**) different validation learning rates using moving average.

Figure 9 compares the learning loss and convergence rates for different batch sizes and learning rates, respectively. Batch size and learning rate are important hyperparameters in model training, and various studies related to the effect of batch size and learning rate on model training have been conducted [60,61,62]. Masters and Luschi [60] showed that higher test accuracy can be obtained with a small batch size by changing the batch size while fixing the learning rate. In addition, the results of fixing the batch size and changing the learning rate showed that when a small batch is used, stable learning is possible over a wider range of learning rates. In addition, Keskar et al. [63] showed that the use of a large batch size increases the likelihood of convergence to a sharp minimum of the training function, which lowers the generalization performance. Therefore, we evaluated the optimal batch size and learning rate to optimize the proposed model.

Figure 9a–c show the evaluation of different batch sizes, and Figure 9d–f show the evaluation of different learning rates. In training and validation loss, as the batch size increased, the convergence speed became slower, and when the batch size was four, it showed the fastest convergence speed. A moving average strategy was used to better discern the visualization as smooth. Figure 9 illustrates how the smoothed value was displayed as a curve and the real value was expressed as the shadow portion of the image for the proposed model’s training. The uneven fluctuation of the real value caused by the small batch size was also addressed, and smoothing was performed to improve the understanding of the curve. In the case of learning rate, there was no training at 0.1 and 0.001, and 0.1 showed a tendency to diverge. On the other hand, among the remaining three learning rates, the 0.0001 value resulted in the fastest convergence speed. Thus, the proposed model exhibits the fastest convergence rate for the collected dataset, with a batch size of four and a learning rate of 0.0001.

### 5.2. Optimization

Hyperparameters that affect training are typically used to determine optimal model updates (e.g., batch size and learning rate). Prior to decreasing the error or loss function caused by the difference between the actual and predicted values, the optimizer must update the weight parameters repeatedly with different weights. However, selecting the right optimizer for the best model training might be challenging. To increase the prediction accuracy and learning rate, training an ideal model is crucial. To determine the model that fits the data the best, we employed the optimization models SGD, RMSprop, Adam, Nesterov-accelerated Adam (Nadam), AdaMax, AdaGrad, and AdaDelta, which are the models most often used for deep learning neural network training.

In comparison to other deep learning neural networks, the SGD [59] optimization strategy is quicker and simpler to train because it eliminates duplication by performing one update at a time. The objective function has considerable fluctuations when the frequent update method with high variance is used, and these fluctuations then have the ability to shift the parameters to new and improved local minima. Because of the ongoing overshooting of SGD, convergence to an accurate minimum is challenging. AdaGrad [57] is a gradient-based optimization approach that adjusts the learning rate of parameters to perform larger updates on repeatedly occurring parameters, while performing fewer updates on less frequently occurring parameters. This approach is suitable for processing sparse data and significantly improves the robustness of SDG optimization [59]. The AdaDelta [56] optimization approach is an extended version of AdaGrad optimization that reduces the learning rate aggressively and monotonically, with parameters varying the learning rates and the learning process stopping after a particular point. The RMSprop [55] optimization approach was developed to overcome the rapidly decreasing learning rate of AdaGrad. It is an adaptive learning rate method, and uses variable learning rates that vary with the results for each sample of each iteration. The Adam [58] optimization approach was developed based on SDG, AdaDelta, and RMSprop. It dynamically calculates the learning rate for each sample of the dataset based on parameters to be used as adaptive optimization approaches with limited memory. The optimization strategy used by Nadam is almost identical to that used by Adam, with the slight difference being that Adam’s and Nesterov’s momentums are combined in Nadam to replace the flat momentum, which greatly improves its performance. The AdaMax [59] optimization approach is an extended approach to Adam optimization that consists of a simpler constraint than the parameter update size of Adam optimization, resulting in stable weight update rules.

**Figure 10:**
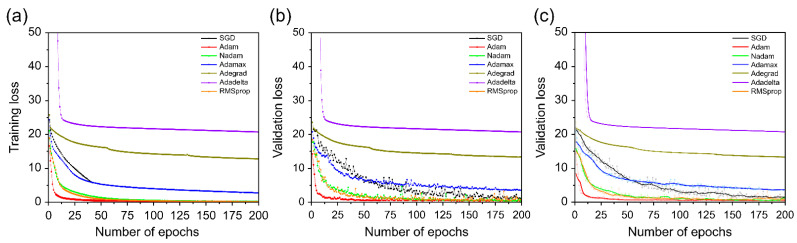
Loss curves comparing various optimizers. (**a**) Training loss; (**b**) validation loss; (**c**) validation loss using moving average.

We compared the training results using a Bi-GRU classifier for the optimization approach of the proposed model. Figure 10 shows the loss curve of learning and validation of the optimization approach. Among the seven optimization techniques, Adam shows the fastest learning process and convergence rate, which means that Bi-GRU classifiers were trained more successfully than other optimization approaches. Conversely, the AdaDelta optimization approach exhibits the lowest learning rate. Consequently, in the optimization approach following batch size and learning rate, Adam was adopted as the optimal approach best suited for training lip-based classification by the proposed model.

### 5.3. Performance and Accuracy

**Figure 11:**
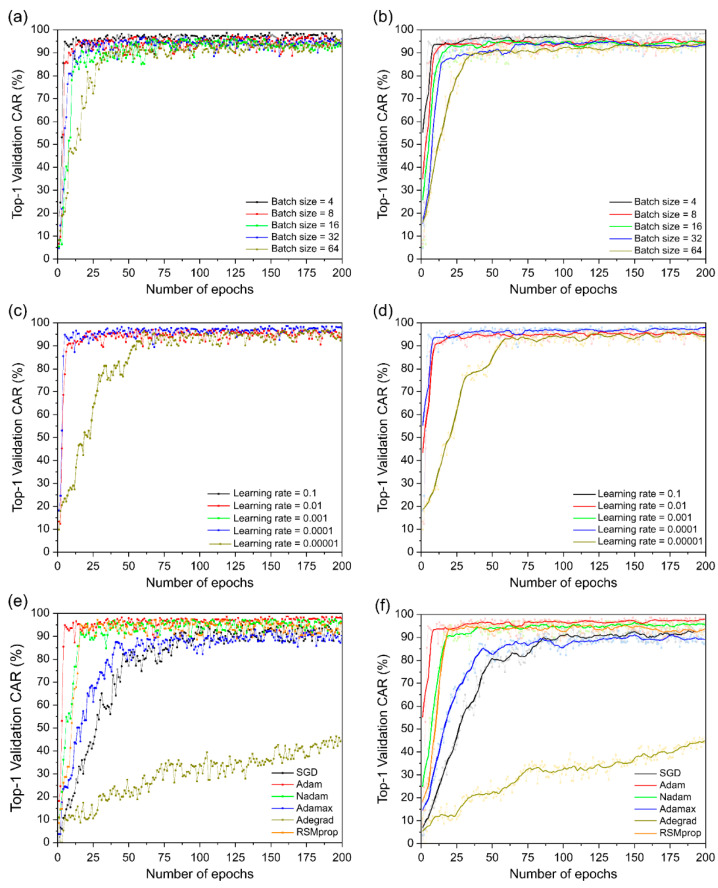
Training steps for character accuracy rate (CAR) comparing different batch sizes, learning rates, and optimizers: (**a**) Different batch sizes; (**b**) different batch sizes using moving average; (**c**) different learning rates; (**d**) different learning rates using moving average; (**e**) different optimizers; (**f**) different optimizers using moving average.

The performance evaluation results of the proposed model are presented in Figure 11 and Table 4. The proposed model obtained the best results, with a CAR of 98.698% at four, the smallest size among different batch sizes, and the CAR decreased as the batch size increased (Figure 11a,b and Table 4). At different learning rates, 0.0001 yielded the best performance, and the remaining 0.01 and 0.0001 yielded similar performances (Figure 11c,d). However, at 0.1 and 0.001, they were excluded from the performance evaluation owing to divergence in the process of training the model. Among the seven optimization approaches, Adam optimization yielded the highest results, followed by Nadam and RMSprop with similar performance (Figure 11e,f). In addition, SGD and AdaMax showed good performance in that order, and AdaGrad showed low performance at 46.392%. AdaDelta was excluded from the performance evaluation because it diverged during the training process of the model. Thus, the proposed model exhibited the best performance when trained with a batch size of four, a learning rate of 0.0001, and the Adam optimization approach.

### 5.4. Confusion Matrix

**Figure 12:**
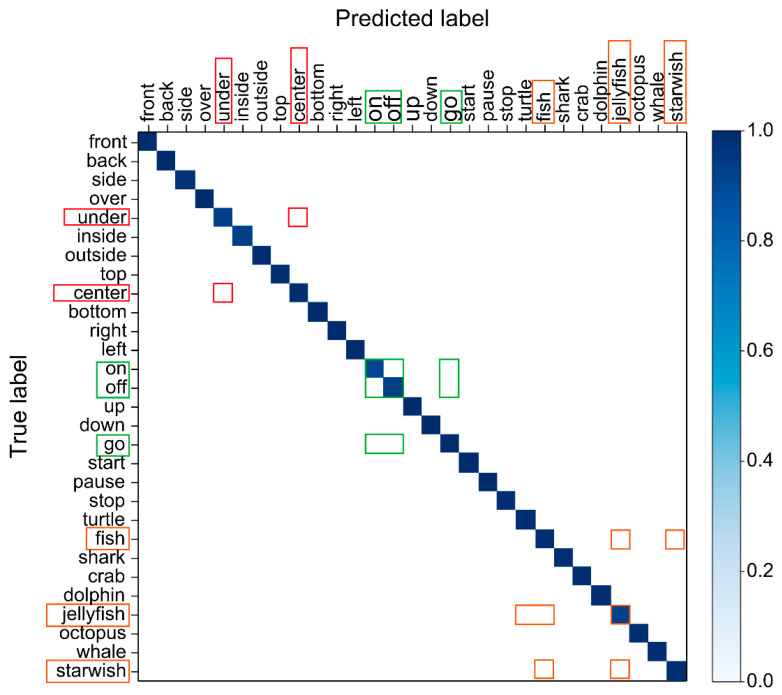
Confusion matrix of the model that proposed many misclassifications out of 53 classes.

**Figure 13:**
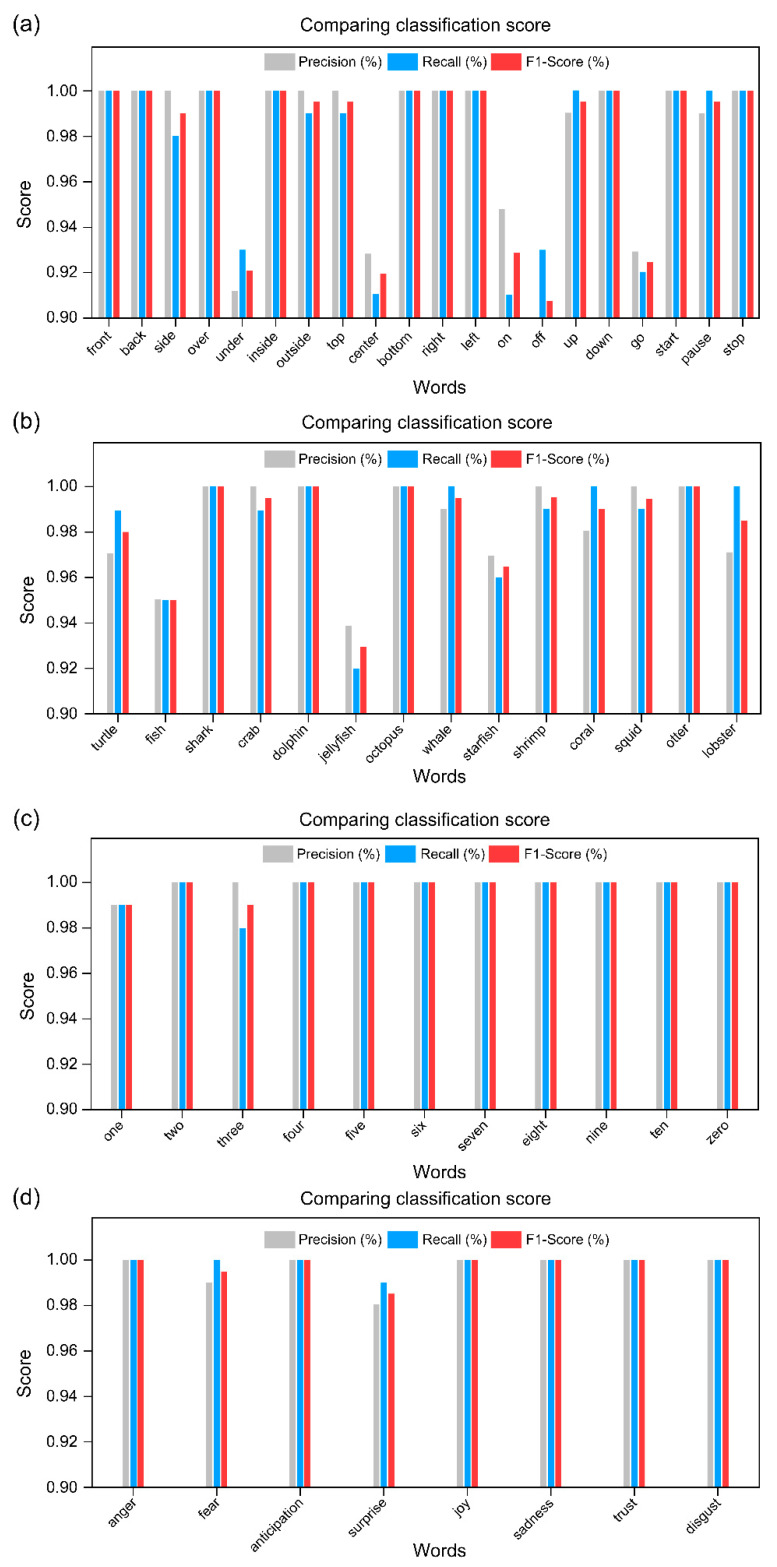
Comparing classification scores of the proposed model trained on 53 classes. (**a**) Control commands; (**b**) marine life; (**c**) number; (**d**) emotion.

**Figure A1:**
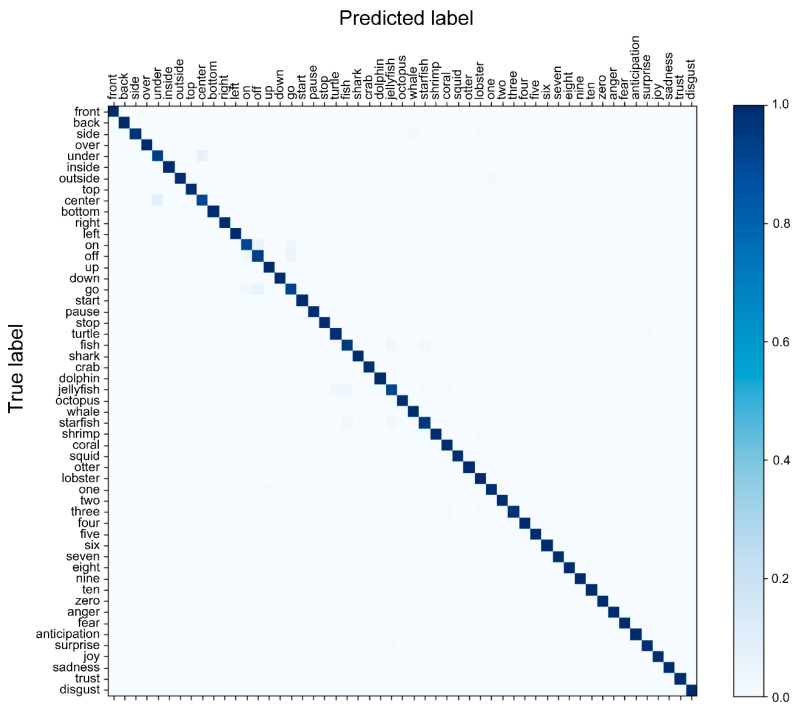
Confusion matrix of the proposed model trained on 53 classes.

The performance of the proposed model was evaluated using 30% of the dataset as a validation sample. The results are shown in Figure A1 and Figure 12 as confusion matrices and Figure 13 as a classification report. The average (mean) precision of each of the proposed models was 0.9870%, recall was 0.9869%, and F1-score was 0.9869% (shown in Table A2). In the classified recognition results, misclassification occurred in “go”, “on”, and “off”, where the mouth was opened only to a small degree and the duration of utterance was shortest. Further, a small misclassification occurred in “center” and “under”, and “fish”, “jellyfish”, and “starfish”, which have similar utterance endings. As reported by Kaburagi et al. [1], this is a problem that occurs because of the similar lip shape at the beginning and end of speech, but it is not a factor that has a great influence on the overall performance evaluation. Based on our evaluation of the proposed model, it is clear that it can help overcome technical obstacles to practical implementation.

### 5.5. Performance and Accuracy

**Figure 14:**
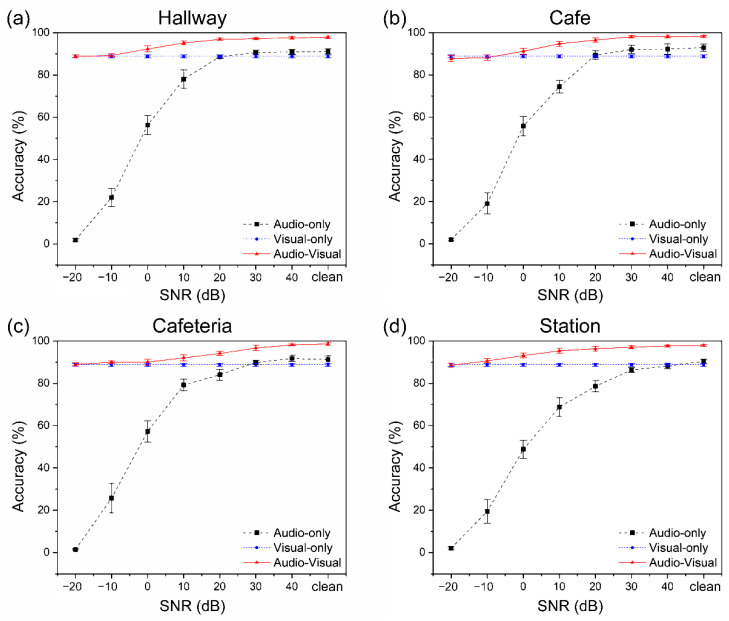
Standard variation of the identification accuracy rate across four noise situations. Standard deviation is shown by error bars. The audio recognition result is shown by the black (single-modal recognition) line, the visual recognition result is represented by the blue (single-modal recognition) line, and the audiovisual recognition result is represented by the red (multimodal recognition) line. The identification outcome in a true experimental setting is called clean SNR.

Figure 8 shows the actual experimental scene where participants interacted using their speech in a virtual aquarium environment, and Figure 14 and Table A3 show the performance of each recognizer at different SNR (dB) levels considering the case of four noises. In Figure 14a, which was evaluated at different SNR levels considering the case of corridor noise, it was 91.14% ± 1.24% in clean, and the highest accuracy was 90.88% ± 1.08% in 40 dB. The visual-only accuracy for various SNR levels was 88.79% ± 0.73%, and because this value depends only on visual information, it is not affected by the clearance of the audio signal or the SNR level. Participants who participated in the data collection stage did not participate in the performance evaluation of the proposed model, and there were no external factors such as lighting for clear data collection during data collection. In addition, in order not to control external factors, the experiment was carried out naturally without limitations of variables (such as mouth shape during pronunciation by participants or mouth shadow according to lighting). The multimodal approach using both auditory and visual signals was 97.87% ± 0.62%, which improved recognition rates by 6.73% and 9.08%, respectively, compared to recognition using only auditory and visual signals. As a result, speech may be inferred even in the presence of background noise by utilizing a mix of sound and visual information.

Based on the previous results, different noises were synthesized and compared for the three components. For the results in Figure 14b and Table A3b, the performance evaluation was performed by synthesizing cafe noise at various SNR levels, and only audio was used for recognition at the clean level in 92.90% ± 1.64% of cases. On the other hand, when audio and visual information were used together for recognition, it was 98.17%± 0.52%, an improvement of 5.27% compared to when only audio was used. Figure 14c,d consist of noise from two different environments: a cafeteria and a subway station. The cafeteria is a busy office cafeteria environment, and the subway is the noise of the main transit area of a crowded subway station. When only audio is used, the recognition rates are 91.26% ± 1.69% and 90.37% ± 1.10%, respectively; and when audio and visual are used together, the recognition rates are 98.60% ± 0.70% and 97.85% ± 0.51%, respectively. The recognition rates were improved by 7.34% and 7.48%, respectively, compared to the case where only audio was used.

**Figure 15:**
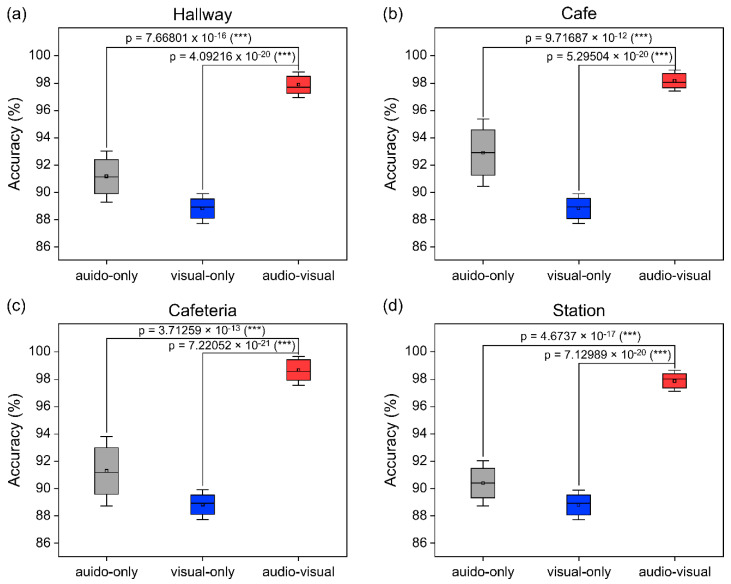
Average recognition accuracy rates in four noisy scenarios. The error bars show the standard deviation. *t*-tests with statistical significance between each group are indicated by asterisks (* for *p* < 0.05, ** for *p* < 0.01, *** for *p* < 0.001). The gray (single-modal recognition), blue (single-modal recognition), and red (multimodal recognition) boxes show the results of auditory-only, visual-only, and audiovisual recognition, respectively.

The statistical significance of the three-group t-test for four noise settings is shown in Figure 15 for each noise environment. In all the noise environments, the recognition rate was improved by 6.04% on average in the case of using both audio and visual compared to the case of using only audio. On the other hand, the recognition rate was improved by 9.33% compared to the case where only vision was used. Additionally, the standard deviation was reduced when audio and visual information were used together, compared to when only audio was used, and a similar recognition rate for repeated experiments was also observed.

## 6. Discussion

Recently, owing to the exponential increase in large-capacity data-processing capability, speech recognition-based interactive edutainment systems have become more widespread. Along with gesture interaction, speech interaction is used in various applications, and more difficult acoustic scenarios have to be considered than in the past for extensive practical applications, taking into account the challenging issue of adequate noise management for varied settings. We conducted a study on speech interaction in a virtual aquarium considering various scenarios.

In this study, an end-to-end visual speech recognition-based interaction system for speech interaction in a virtual aquarium environment was proposed. Recognized words were vectored by combining pretrained word embedding with Microsoft API, which showed the best performance and dense dispersion in previous studies [45]. Feature vectorization was performed on the image sequence input through video via the visual processing module, and word vectorization was combined to output the predicted word. For the optimization of the proposed model, the performance was evaluated using different batch sizes, learning rates, and optimization approaches. In addition, performance evaluation was performed by synthesizing the data used in the actual evaluation and four noise types using different SNR levels.

The system performance in terms of SNR was evaluated using four sets of synthesized data associated with four noise environments along with other evaluation data. Compared to the speech recognition system using only audio, which has a large standard deviation in the four noise environments, the system using visual information showed small standard deviation and superior performance. Furthermore, in the case of clean, the average recognition rate was 91.42% when only speech was used in the four noise environments, whereas when audio and visual information were used together, the recognition rate was improved by 6.7% to 98.12%.

The utilization of multimodal interactions based on visual information is necessary to develop antinoise automated speech recognition (ASR) systems. The proposed system could help patients who have difficulty with noise during conversation. It can also provide an opportunity for people with hearing problems to have a conversation. However, it is difficult to apply this technique to real-world conversation recognition. Therefore, in future studies, we will consider expanding the system’s ability to identify non-keyword-centric phrases. Additionally, by displaying an actual virtual aquarium in a science museum, we will assess the efficiency of the proposed model in a real environment and create a reliable portable gadget for real-world usage.

## 7. Conclusions

By combining visual information with the existing speech interaction-based edutainment system, a new visual information-based speech recognition system that is robust to noise was applied to a virtual aquarium and demonstrated. The proposed system combines audio and visual information, and for optimization, the performance was evaluated considering three factors (batch size, learning rate, and optimization approach), and the performance was further evaluated in four noise environments. It was shown that visual information contributed to the improvement of speech recognition by using visual information, unlike the existing method that used only speech to interact. To achieve consistent and high performance in diverse noise conditions, our technique blends visual speech recognition, a technology that can enhance speech recognition systems, with current cloud-based speech recognition systems. This method offers the potential for real-world speech recognition applications in noisy locations. It can be utilized for speech interaction in venues such as museums and scientific centers that have significant amounts of interior noise and noise from visitors, and it also has the potential for use in diverse applications that employ voice recognition in noise, such as IoT and robot applications.

## Figures and Tables

**Table 1:** Collected speech commands datasets: (a) control commands; (b) marine life; (c) numbers; (d) emotions.

**(a) Control Commands (20)**				
front	back	side	over	under
inside	outside	top	center	bottom
right	left	on	off	up
down	go	start	pause	stop
**(b) Marine Life (15)**				
turtle	fish	shark	crab	dolphin
jellyfish	octopus	whale	starfish	shrimp
coral	squid	otter	lobster	
**(c) Numbers (11)**				
one	two	three	four	five
six	seven	eight	nine	ten
zero				
**(d) Emotions (8)**				
anger	fear	anticipation	surprise	joy
sadness	trust	disgust		

**Table 2:** Confusion matrix for two classes.

		Predicted Class	
		Actual Positive	Actual Negative
Actual Class	Predictive Positive	True Positive (TP)	False Positive (FP)
	Predictive Negative	False Negative (FN)	True Negative (TN)

**Table 3:** Noise database structure: categories and recordings conducted in each category.

	Category	Place	Environment
(a)	Office	Hallway	Hallway inside an office building, with individuals and groups passing by occasionally
(b)	Public	Cafeteria	Busy office cafeteria
(c)		Station	Main transfer area of a busy subway station
(d)	Street	Cafe	Terrace of a cafe at a public square

**Table 4:** Performance of different batch sizes, learning rates, and optimizers on the collected dataset.

Batch Size	Top-1 CAR (%)	Learning Rate	Top-1 CAR (%)	Optimizer	Top-1 CAR (%)
4	98.698	0.1	-	SGD	94.867
8	97.663	0.01	97.301	Adam	98.698
16	97.657	0.001	-	Nadam	97.500
32	96.972	0.0001	98.698	AdaMax	92.953
64	96.126	0.00001	97.095	AdaGrad	46.392
				AdaDelta	-
				RMSprop	97.323

## Data Availability

The data presented in this study are available on request from the corresponding author. The data are not publicly available due to ethical restrictions.

## Appendix A

The following Table A1, Table A2 and Table A3 and Figure A1 provide (1) the details of the hyperparameters associated with the proposed architecture, (2) the confusion matrix of the proposed model trained on 53 classes, (3) the average word accuracy and standard deviation of the proposed system in four noise environments, and (4) the performance in terms of precision, recall, and F1-score, respectively.

**Table A1:** Hyperparameters associated with the proposed architecture.

Layers	Size/Stride/Pad		Visual	Audio
			Output Size	
Input Layer	-		40 × 100 × 50 × 3	
3D Conv Layer	[3 × 5 × 5]/(1, 2, 2)/(1, 2, 2)		40 × 50 × 25 × 64	1500
3D Max Pooling	[1 × 2 × 2]/(1, 2, 2)		40 × 50 × 13 × 64	
Densely Connected 3D CNN	[3 × 1 × 1] 3D Conv	(×6)	40 × 25 × 13 × 96	
	[3 × 3 × 3] 3D Conv			
	[3 × 1 × 1] 3D Conv		40 × 12 × 6 × 6	
	[1 × 2 × 2] average pool/(1 × 2 × 2)			
	[3 × 1 × 1] 3D Conv	(×12)	40 × 12 × 6 × 38	
	[3 × 3 × 3] 3D Conv			
	[3 × 1 × 1] 3D Conv		40 × 6 × 3 × 3	
	[1 × 2 × 2] average pool/(1 × 2 × 2)			
	[3 × 1 × 1] 3D Conv	(×24)	40 × 12 × 6 × 38	
	[3 × 3 × 3] 3D Conv			
	[3 × 1 × 1] 3D Conv		40 × 3 × 1 × 1	
	[1 × 2 × 2] average pool/(1 × 2 × 2)			
	[3 × 1 × 1] 3D Conv	(×16)	40 × 3 × 1 × 33	
	[3 × 3 × 3] 3D Conv			
Multiscale 3D CNN (1)	[3 × 5 × 5]/(1, 2, 2)/(1, 2, 2)		40 × 3 × 1 × 32	
Multiscale 3D CNN (2)	[3 × 5 × 5]/(1, 2, 2)/(1, 2, 2)		40 × 3 × 1 × 64	
Multiscale 3D CNN (3)	[3 × 5 × 5]/(1, 2, 2)/(1, 2, 2)		40 × 3 × 1 × 92	
Spatial Attention (1)	[1 × 2 × 2] max pool/(1 × 2 × 2)		40 × 3 × 1 × 32	
	[1 × 2 × 2] average pool/(1 × 2 × 2)			
	[3 × 7 × 7]/(1, 2, 2)/(1, 2, 2)			
Spatial Attention (2)	[1 × 2 × 2] max pool/(1 × 2 × 2)		40 × 3 × 1 × 64	
	[1 × 2 × 2] average pool/(1 × 2 × 2)			
	[3 × 7 × 7]/(1, 2, 2)/(1, 2, 2)			
Spatial Attention (3)	[1 × 2 × 2] max pool/(1 × 2 × 2)		40 × 3 × 1 × 96	
	[1 × 2 × 2] average pool/(1 × 2 × 2)			
	[3 × 7 × 7]/(1, 2, 2)/(1, 2, 2)			
Bi-GRU (1)	256		40 × 512	
Bi-GRU (2)	256		40 × 512	
Concatenation	-		40 × 2012	
Dense Layer	27 + blank		40 × 2012	
Softmax			40 × 28	

**Table A2:** Average word accuracy and standard deviation of the proposed system in four noise environments.

SNR (dB)		−20	−10	0	10	20	30	40	Clean
(a) Hallway	A	1.84% ± 0.62%	21.98% ± 4.30%	56.26% ± 4.63%	78.01% ± 4.42%	88.60% ± 0.88%	90.59% ± 1.07%	90.88% ± 1.08%	91.14% ± 1.24%
	V	88.79% ± 0.73%	88.79% ± 0.73%	88.79% ± 0.73%	88.79% ± 0.73%	88.79% ± 0.73%	88.79% ± 0.73%	88.79% ± 0.73%	88.79% ± 0.73%
	AV	88.84% ± 0.65%	89.27 ± 0.70%	92.33 ± 1.41%	95.19 ± 0.96%	96.84 ± 0.58%	97.20% ± 0.44%	97.58% ± 0.54%	97.87% ± 0.62%
(b) Cafe	A	1.99% ± 0.28%	19.05% ± 4.99%	55.77% ± 4.54%	74.42% ± 3.05%	89.34% ± 2.04%	92.04% ± 2.01%	92.23% ± 2.50%	92.90% ± 1.64%
	V	88.79% ± 0.73%	88.79% ± 0.73%	88.79% ± 0.73%	88.79% ± 0.73%	88.79% ± 0.73%	88.79% ± 0.73%	88.79% ± 0.73%	88.79% ± 0.73%
	AV	87.70% ± 1.51%	88.17% ± 1.29%	91.16% ± 1.43%	94.70% ± 1.15%	96.48% ± 0.93%	98.02% ± 0.61%	98.10% ± 0.65%	98.17% ± 0.52%
(c) Cafeteria	A	1.44% ± 0.31%	25.74% ± 6.92%	57.30% ± 5.00%	79.23% ± 2.81%	84.06% ± 2.57%	89.90% ± 1.05%	91.76% ± 1.54%	91.26% ± 1.69%
	V	88.79% ± 0.73%	88.79% ± 0.73%	88.79% ± 0.73%	88.79% ± 0.73%	88.79% ± 0.73%	88.79% ± 0.73%	88.79% ± 0.73%	88.79% ± 0.73%
	AV	88.92% ± 0.79%	89.94% ± 0.57%	90.07% ± 1.36%	92.05% ± 1.43%	94.13% ± 1.04%	96.63% ± 1.30%	98.17% ± 0.46%	98.60% ± 0.70%
(d) Station	A	2.08% ± 0.69%	19.49% ± 5.64%	48.84% ± 4.30%	68.79% ± 4.46%	78.61% ± 2.67%	86.31% ± 1.17%	88.23% ± 1.34%	90.37% ± 1.10%
	V	88.79% ± 0.73%	88.79% ± 0.73%	88.79% ± 0.73%	88.79% ± 0.73%	88.79% ± 0.73%	88.79% ± 0.73%	88.79% ± 0.73%	88.79% ± 0.73%
	AV	88.57% ± 0.99%	90.64% ± 0.96%	93.10% ± 1.12%	95.34% ± 1.20%	96.34% ± 1.05%	97.06% ± 0.63%	97.67% ± 0.47%	97.85% ± 0.51%

**Table A3:** Precision, recall, and F1-score performance of in four noise environments. (a) control commands; (b) marine life; (c) numbers; (d) emotions.

**(a) Control Commands**				**(b) Marine Life**			
**Word**	**Precision**	**Recall**	**F1-Score**	**Word**	**Precision**	**Recall**	**F1-Score**
front	1.0000	1.0000	1.0000	turtle	0.9706	0.9900	0.9802
back	1.0000	1.0000	1.0000	fish	0.9500	0.9500	0.9500
side	1.0000	0.9800	0.9899	shark	1.0000	1.0000	1.0000
over	1.0000	1.0000	1.0000	crab	1.0000	0.9900	0.9950
under	0.9118	0.9300	0.9208	dolphin	1.0000	1.0000	1.0000
inside	1.0000	1.0000	1.0000	jellyfish	0.9388	0.9200	0.9293
outside	1.0000	0.9900	0.9950	octopus	1.0000	1.0000	1.0000
top	1.0000	0.9900	0.9950	whale	0.9901	1.0000	0.9950
center	0.9286	0.9100	0.9192	starfish	0.9697	0.9600	0.9648
bottom	1.0000	1.0000	1.0000	shrimp	1.0000	0.9900	0.9950
right	1.0000	1.0000	1.0000	coral	0.9804	1.0000	0.9901
left	1.0000	1.0000	1.0000	squid	1.0000	0.9900	0.9950
on	0.9479	0.9100	0.9286	otter	1.0000	1.0000	1.0000
off	0.8857	0.9300	0.9073	lobster	0.9709	1.0000	0.9852
up	0.9901	1.0000	0.9950				
down	1.0000	1.0000	1.0000				
go	0.9293	0.9200	0.9246				
start	1.0000	1.0000	1.0000				
pause	0.9901	1.0000	0.9950				
stop	1.0000	1.0000	1.0000				
**(c) Numbers**				**(d) Emotions**			
**Word**	**Precision**	**Recall**	**F1-Score**	**Word**	**Precision**	**Recall**	**F1-Score**
one	0.9900	0.9900	0.9900	anger	1.0000	1.0000	1.0000
two	1.0000	1.0000	1.0000	fear	0.9901	1.0000	0.9950
three	1.0000	0.9800	0.9899	anticipation	1.0000	1.0000	1.0000
four	1.0000	1.0000	1.0000	surprise	0.9802	0.9900	0.9851
five	1.0000	1.0000	1.0000	joy	1.0000	1.0000	1.0000
six	1.0000	1.0000	1.0000	sadness	1.0000	1.0000	1.0000
seven	1.0000	1.0000	1.0000	trust	1.0000	1.0000	1.0000
eight	1.0000	1.0000	1.0000	disgust	1.0000	1.0000	1.0000
nine	1.0000	1.0000	1.0000				
ten	1.0000	1.0000	1.0000				
zero	1.0000	1.0000	1.0000				
